# Supplementary material for: Obtaining free USArray data by multi-dimensional seismic reconstruction
Source: Nat Commun. 2019 Sep 30;10:4434. doi: 10.1038/s41467-019-12405-0 (PMC6768879; doi:10.1038/s41467-019-12405-0)
Supplement: Supplementary file 1 — Supplementary Information [file 41467_2019_12405_MOESM1_ESM.pdf]

# Supplementary Information for: Obtaining free USArray data by multi-dimensional seismic reconstruction

Chen et al.

## Supplementary Note 1

We test the performance of the rank-reduction method in data reconstruction and noise suppression using another 3D synthetic example (Supplementary Figure 1(a)). The data consists of 500 time samples, and 60 samples in both spatial directions. This synthetic data is specifically designed to mimic the earthquake arrivals in realistic situation. There are three phase arrivals in the data, which resembles several key features of the real earthquake waveforms including firstly the highly-curved wavefronts, secondly the large spatiotemporal amplitude variations, and thirdly the weak-energy arrivals. Since the rank-reduction method is based on the plane-wave assumption (i.e., a linear phase arrival), the curving wavefront in the data is used to test the effectiveness of the proposed method in the case of non-planar waves. The first phase in the synthetic data is characterized by a constant-amplitude, which resembles the teleseismic body waves incident at steep angle beneath the recording array. The second phase is obtained by perturbing the arrival times and amplitudes of the first arrivals (Supplementary Figure 2), which could represent the delay or advance of wavefields due to strong heterogeneity. The third phase has an amplitude that is only a quarter of that of the first arrival, which simulates the weak phases such as precursory arrivals or crustal converted waves. We add random noise with amplitude of 10% of the maximum amplitude of the signal into the clean data to produce the noisy recordings (Supplementary Figure 1(b)). The missing traces are generated by applying the sampling matrix (Supplementary Figure 1(c)) to the noisy data. Supplementary Figure 1(d) shows the final simulated data with 20% missing traces. The respective data quality metrics signal-to-noise ratio (SNR) for the noisy and incomplete data are 7.51 and 4.64 dB, suggesting a significantly decreased data quality due to the presence of missing traces.

The reconstructed data using global and localized rank-reduction methods are plotted in Supplementary Figures 1(a) and 1(b). The reconstruction errors are plotted in Supplementary Figures 1(c) and 1(d), respectively. The respective SNRs for the global and localized reconstructions are 7.51 dB and 14.38 dB, respectively. We extract a single trace at the location where large curvature exists (Supplementary Figure 4). It is clear that the reconstructed trace using localized rank-reduction method (green) almost fully recovers the input (black; Supplementary Figure 1(b)), whereas an obvious time shift is present in the trace obtained from the global method (blue; see Supplementary Figure 1(b)). The local similarity between the clean and the reconstructed data using the global and localized rank-reduction methods are plotted in Supplementary Figures 1(a) and 1(b). We also investigate the effects of sampling ratios (i.e., the percentage of missing data ) on the reconstruction based on this example. We vary the sampling ratio from 90% to 10% and randomly remove the traces. The reconstruction performance as a function of sampling ratio is shown in Supplementary Figure 6.

## Supplementary Note 2

Let $\mathbf{x}$ denote the local similarity between vectors $\mathbf{v}_1$ and $\mathbf{v}_2$. It can be calculated by:

$$\mathbf{x} = \sqrt{\mathbf{x}_1 \circ \mathbf{x}_2}, \tag{1}$$

where $\circ$ means elementwise product. $\mathbf{x}_1$ and $\mathbf{x}_2$ are temporary variables that are calculated by:

$$\min_{\mathbf{x}_1} \|\mathbf{v}_1 - \mathbf{V}_2\mathbf{x}_1\|_2^2, \tag{2}$$

$$\min_{\mathbf{x}_2} \|\mathbf{v}_2 - \mathbf{V}_1\mathbf{x}_2\|_2^2. \tag{3}$$

Here, $\mathbf{V}_1$ and $\mathbf{V}_2$ are both diagonal operators composed of the elements of $\mathbf{v}_1$, $\mathbf{v}_2$, respectively. Equations 2 and 3 can be solved using shaping regularization[1] as follows:

$$\mathbf{x}_1 = [\lambda_1^2\mathbf{I} + \mathbf{T}(\mathbf{V}_1^T\mathbf{V}_1 - \lambda_1^2\mathbf{I})]^{-1}\mathbf{T}\mathbf{V}_1^T\mathbf{v}_2, \tag{4}$$

$$\mathbf{x}_2 = [\lambda_2^2\mathbf{I} + \mathbf{T}(\mathbf{V}_2^T\mathbf{V}_2 - \lambda_2^2\mathbf{I})]^{-1}\mathbf{T}\mathbf{V}_2^T\mathbf{v}_1, \tag{5}$$

where $\mathbf{T}$ corresponds to a smoothness constraint. $\lambda_1$ and $\lambda_2$ are normally chosen as the least-squares norms of $\mathbf{v}_1$ and $\mathbf{v}_2$, respectively, to enable a fast convergence.

## Supplementary Note 3

We use a weighted interpolation method for the binning process. At each node location, the waveforms from nearby stations that are located within one grid distance are stacked and subsequently assigned to the node, whereas the node remains empty if no neighboring stations is available. We use a grid size with respective dimensions of 1.0 deg and 0.5 deg in latitude and longitude directions, which is comparable to the station spacing of USArray, to minimize the effect of spatial smoothing while preserving small-scale features. Future study would focus on interpolating an arbitrary geometry given a randomly distributed data set. Supplementary Figure 10 shows the sampling matrix after the binning process, with its elements defined as zero or one for empty and data-filled grid points, respectively.

## Supplementary Figures

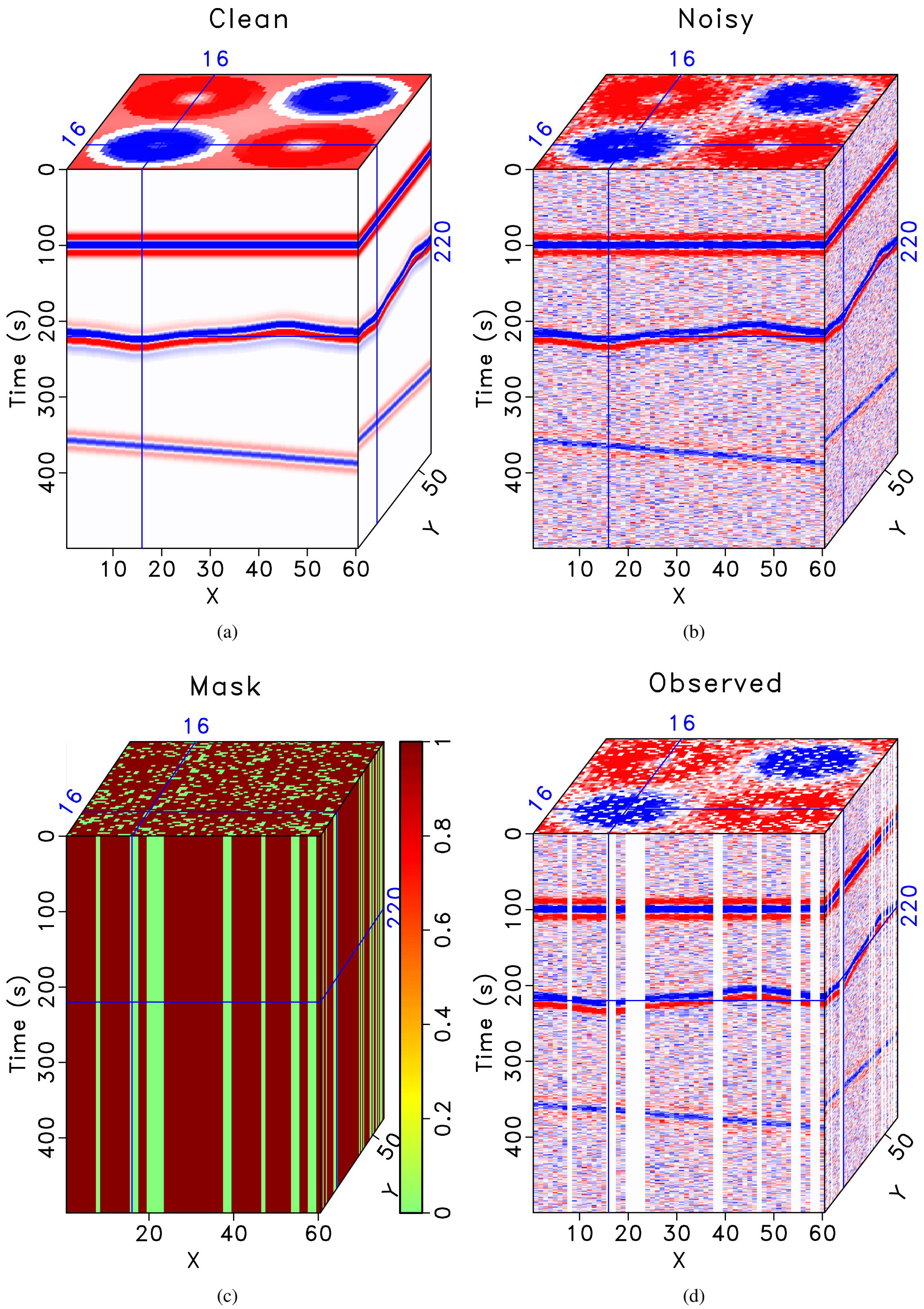


**Supplementary Figure 1.** The relatively simpler synthetic data example. (a) The clean data containing three main seismic phases. The circles at zero time slice indicate the relative time perturbations of the second phase arrivals. The cross-hair marks the location of the sample trace shown in Figure 6. (b) The noisy data with signal-to-noise ratio equal to 7.32 dB. (c) The sampling matrix with a sampling ratio of 80% (d) The final data after applying the sampling matrix to noisy data in (b). The signal-to-noise ratio of the data is 4.64 dB.

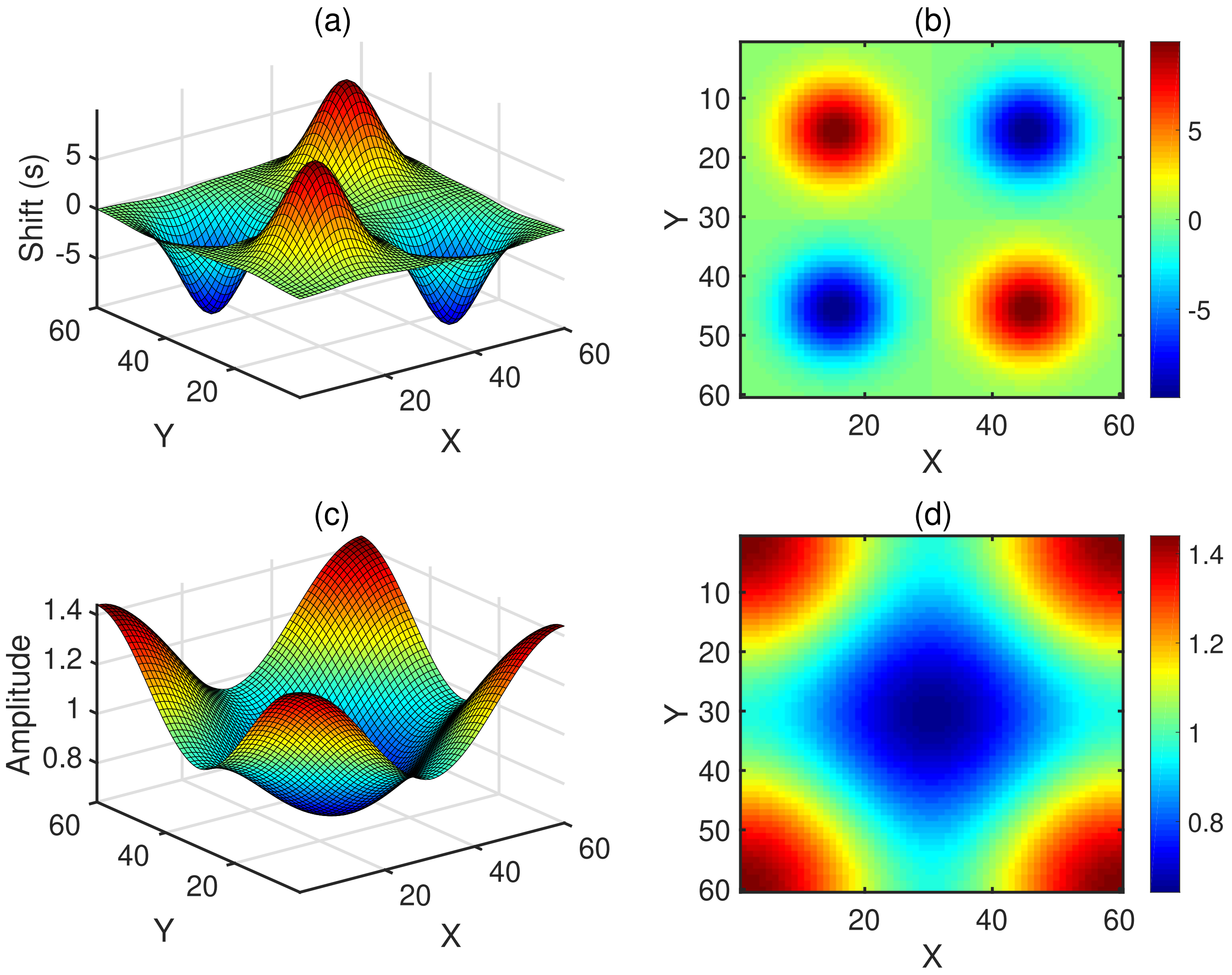


**Supplementary Figure 2.** Time shifts and amplitude variation (for the second event) used in generating the synthetic data. (a) Time shifts in the SURF plot. (b) Time shifts in the GREY plot. (c) Amplitude variation in the SURF plot. (d) Amplitude variation in the GREY plot.

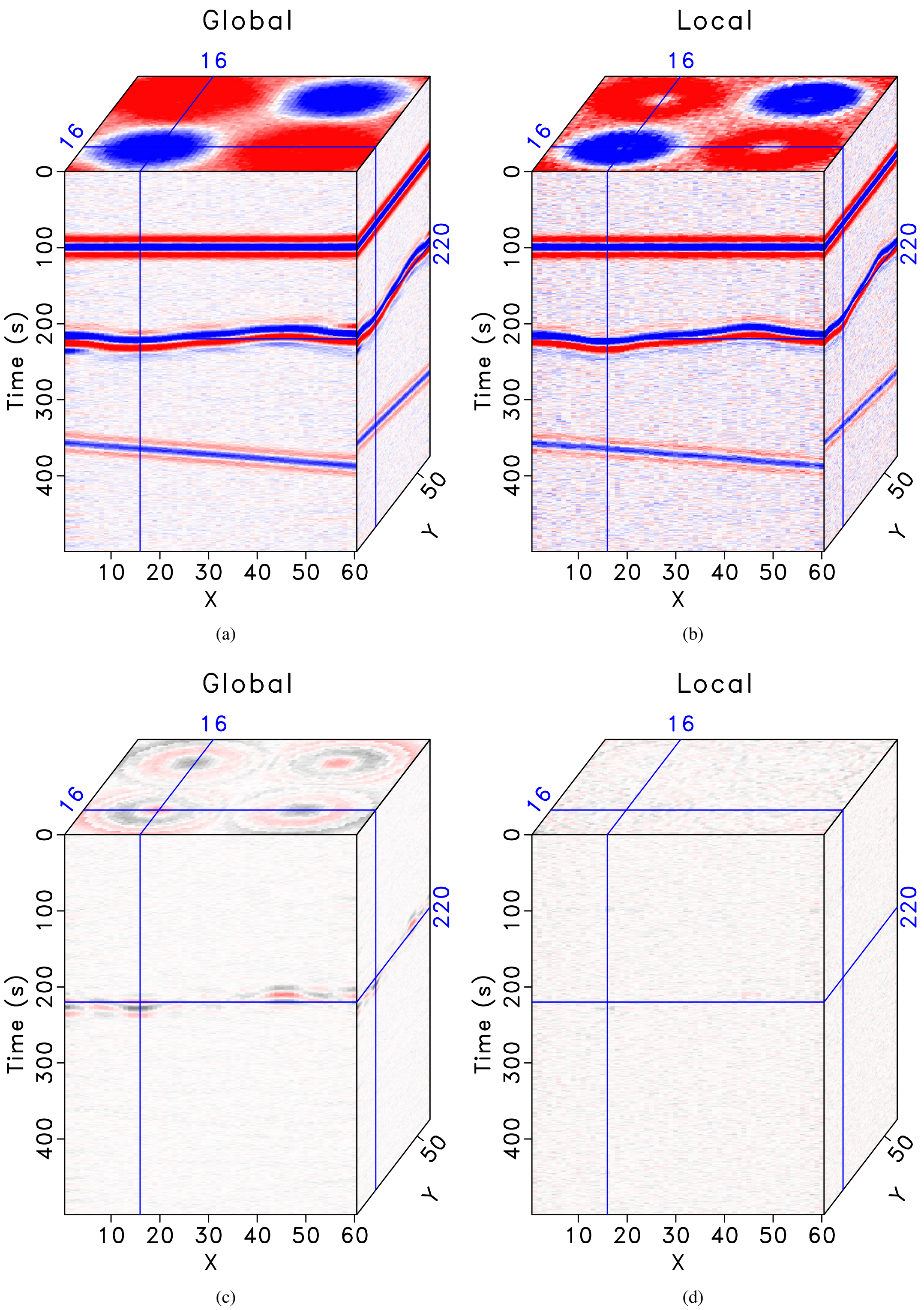


**Supplementary Figure 3.** Synthetic test results. (a) Reconstructed data using the global rank-reduction method. The signal-to-noise ratio of the reconstructed data is 7.51 dB. (b) Reconstructed data using the localized rank-reduction method (signal-to-noise ratio is 14.38 dB). (c) and (d) Reconstruction errors corresponding to (a) and (b).

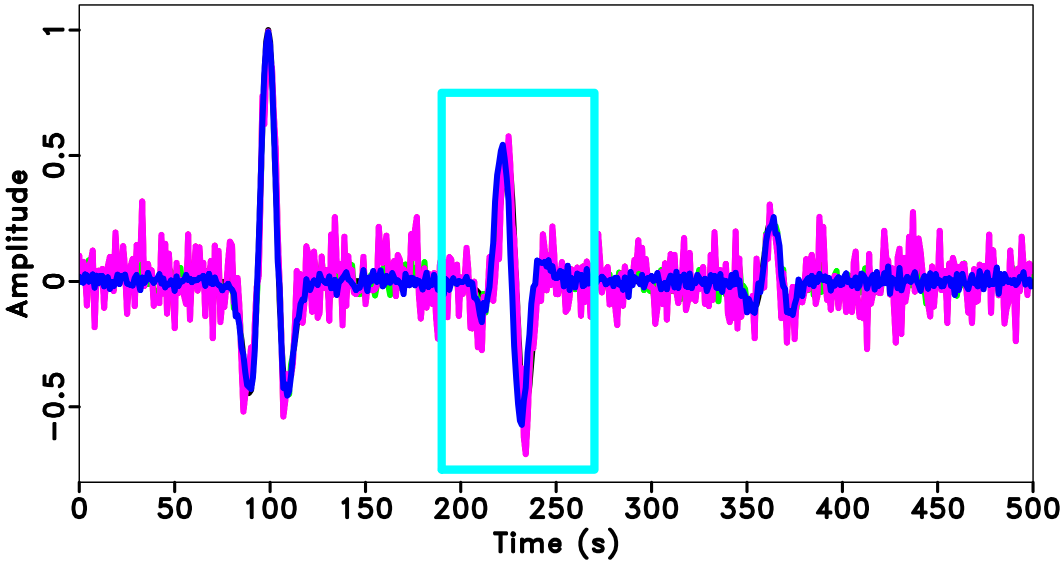


(a)

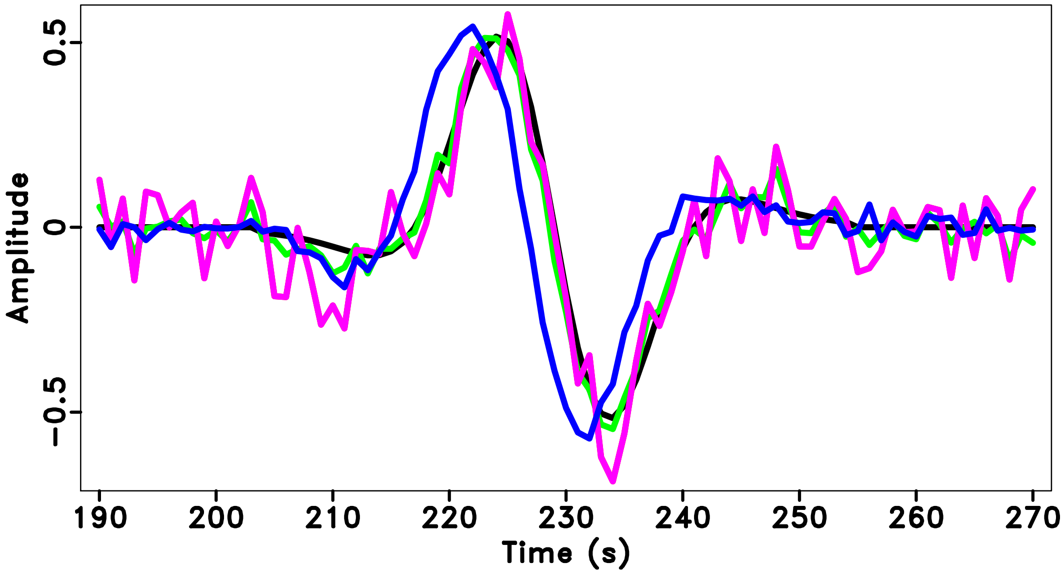


(b)

**Supplementary Figure 4.** Comparison of the single trace amplitude (X=16,Y=16) in different scales. (a) Comparison in the original scale. (b) Comparison in the zoomed-in scale. The black and pink lines represent the clean and noisy data, respectively. The trace processed using global and localized rank-reduction methods are shown by the blue and green lines, respectively.

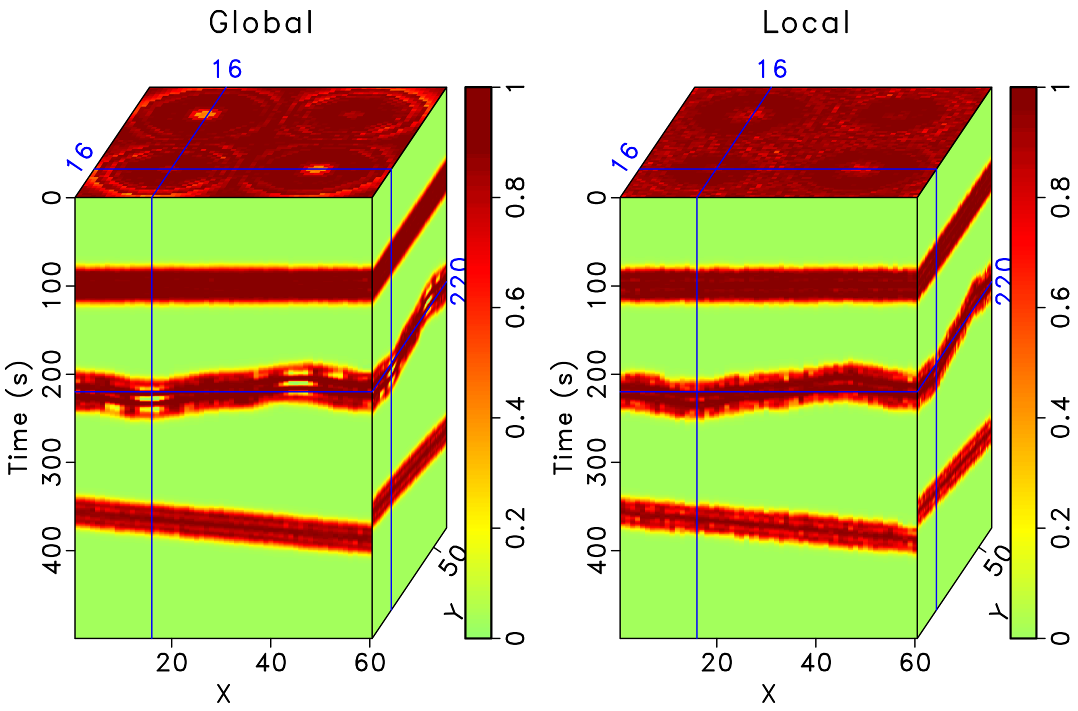


(a) (b)

**Supplementary Figure 5.** A comparison of reconstruction performance in terms of the local similarity metric. (a) Local similarity using the global rank-reduction method. (b) Local similarity using the localized rank-reduction method.

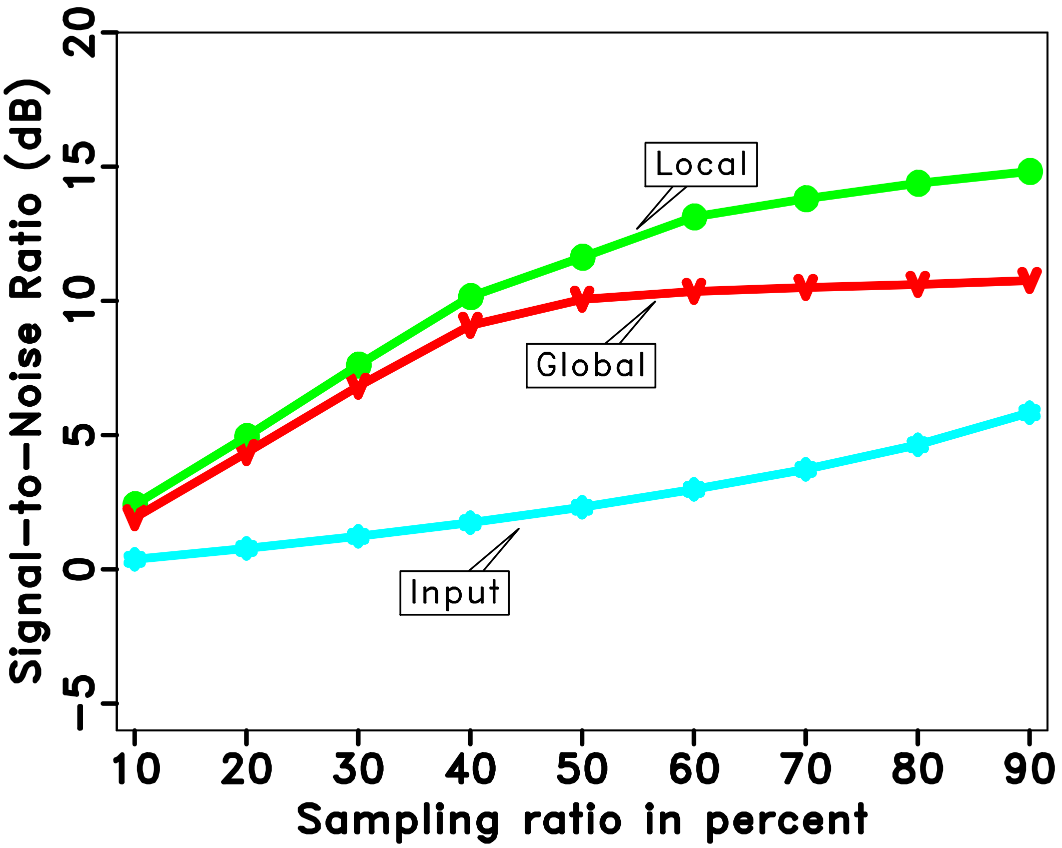


**Supplementary Figure 6.** Reconstruction results at different sampling ratios. The reconstruction performance (signal-to-noise ratio) improves with the increasing sampling ratio. The localized rank-reduction method always outperforms the global algorithm.

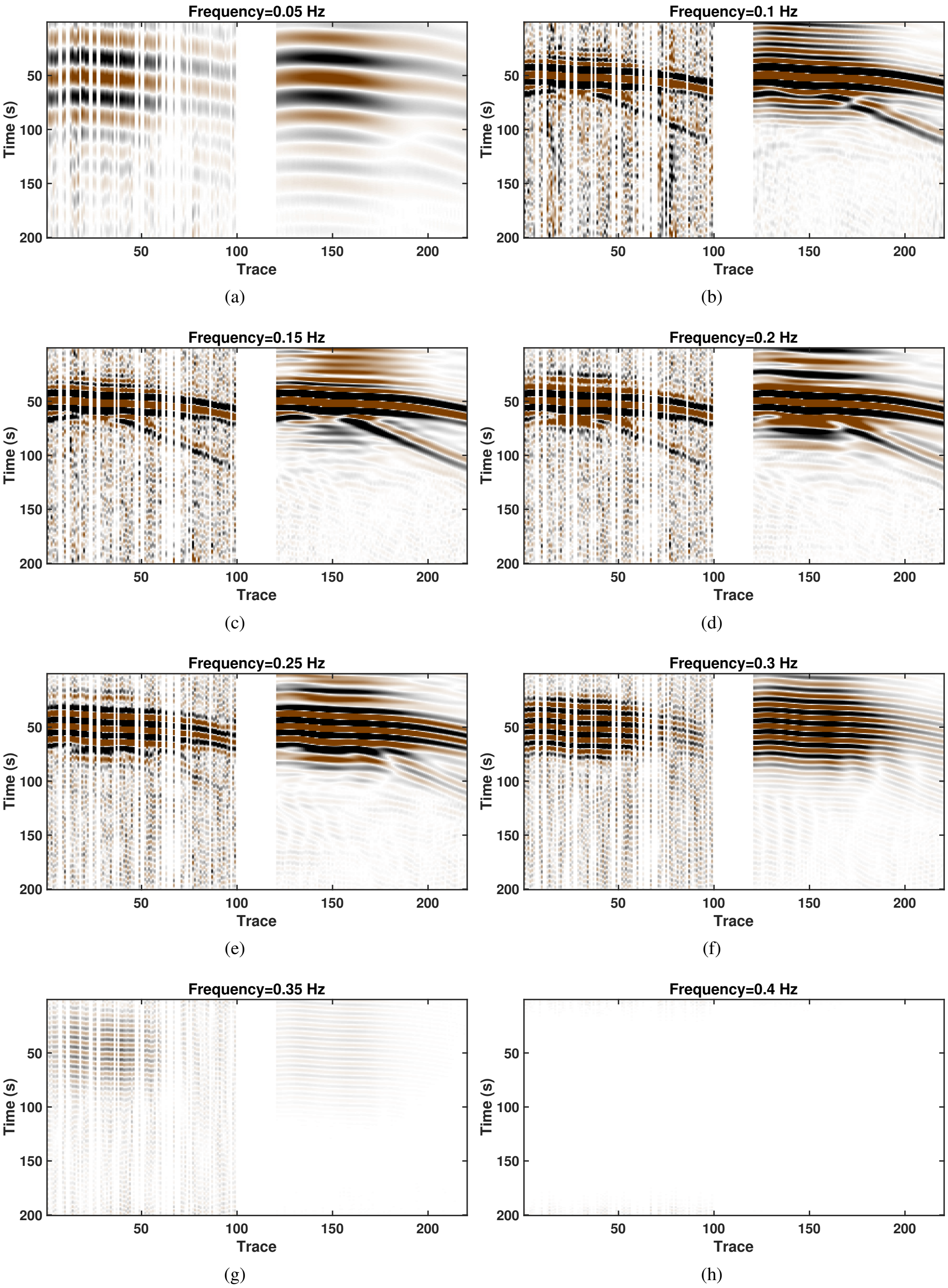


**Supplementary Figure 7.** Reconstruction performance with respect to different frequency contents. (a)-(h) Reconstruction performance for frequencies linearly increased from 0.05 Hz to 0.4 Hz.

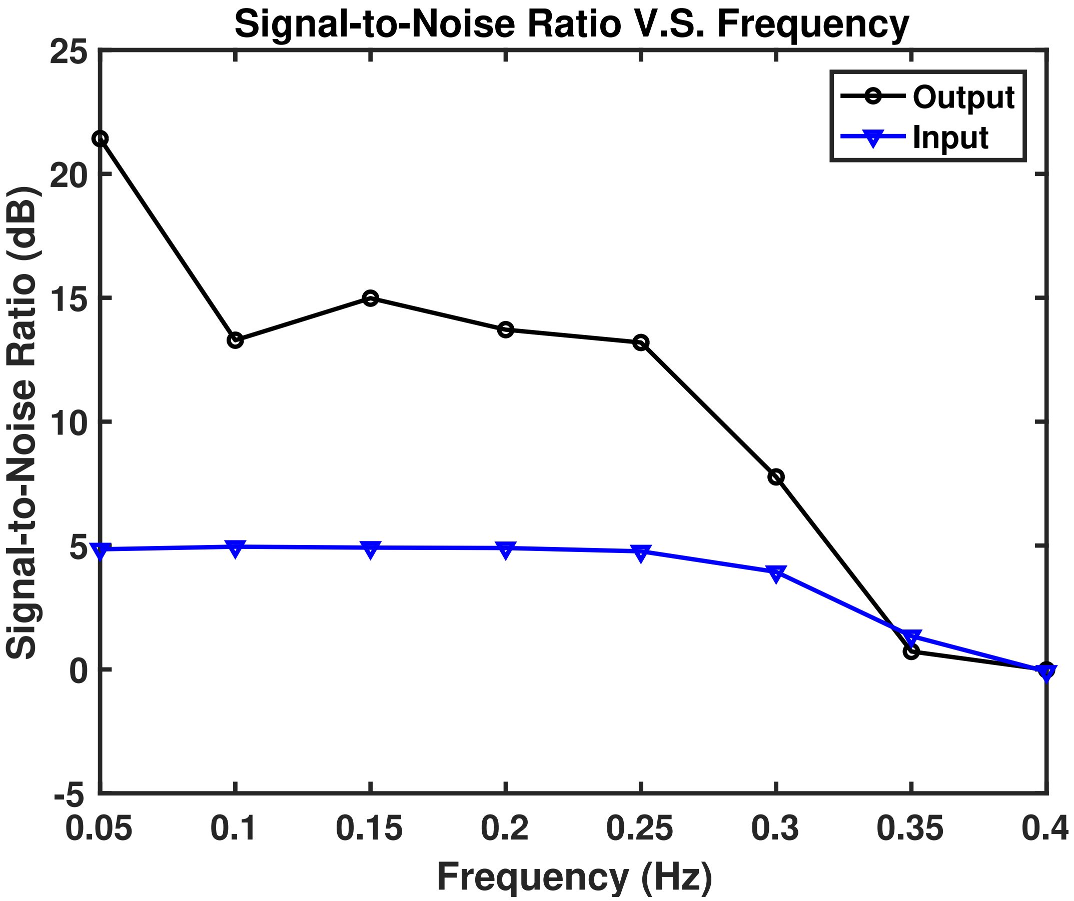


**Supplementary Figure 8.** Diagrams showing the relation between output and input signal-to-noise ratios.

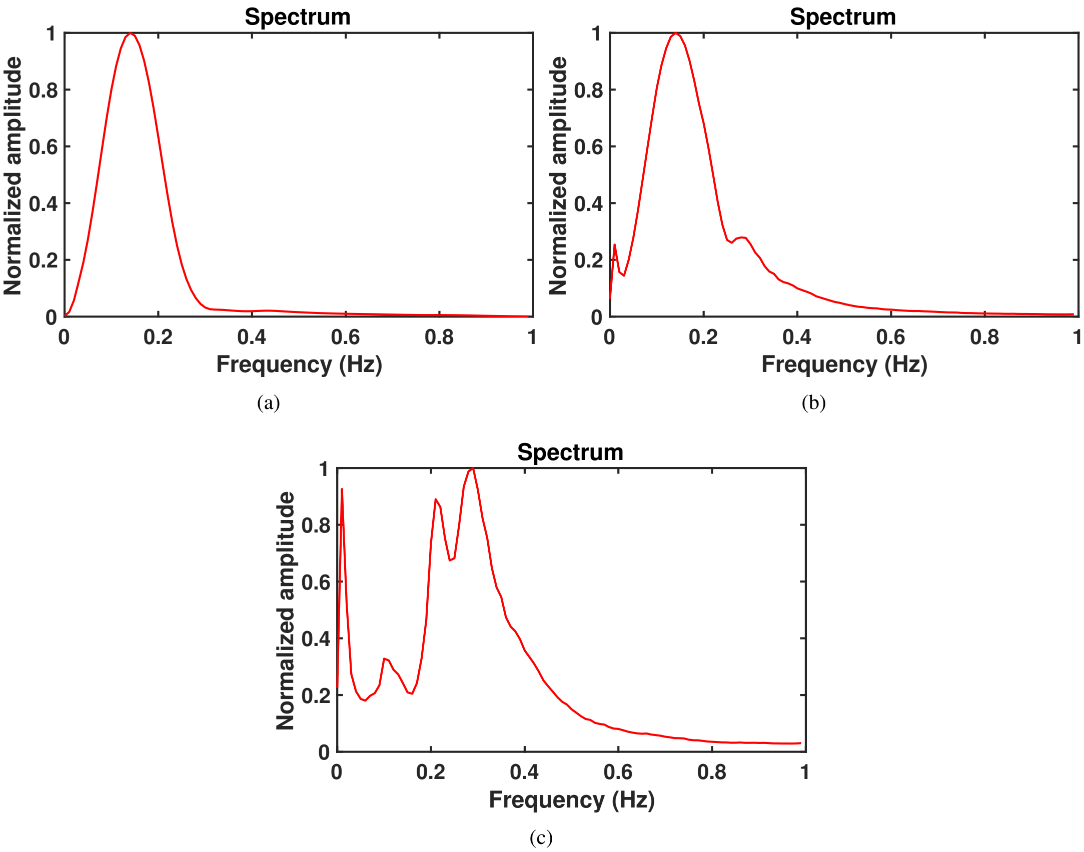


**Supplementary Figure 9.** Spectrum comparison of the synthetic data. (a) Spectrum of the clean data. (b) Spectrum of the observed noisy and incomplete data. (c) Spectrum of the added noise.

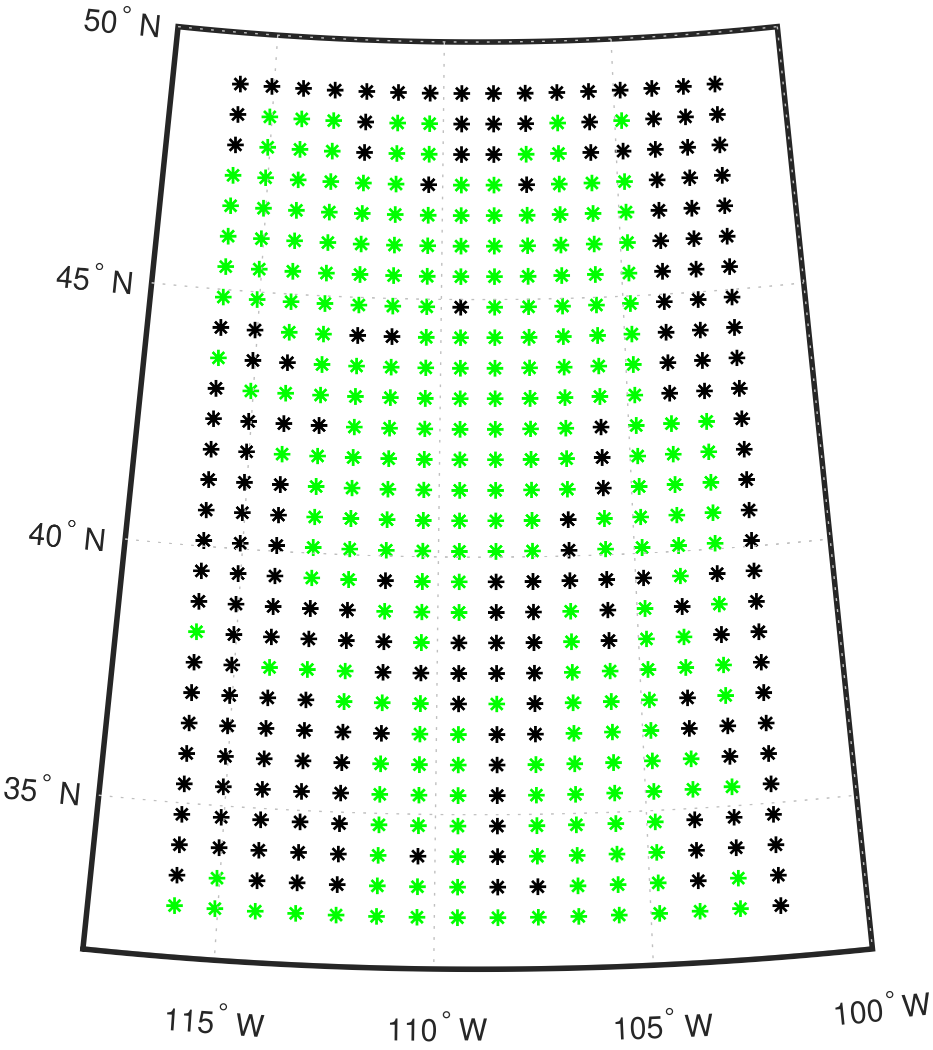


**Supplementary Figure 10.** The sampling matrix. The green and black asterisks denote 1 and 0 in the sampling matrix, respectively. The sampling ratio in this example is 56.5%.

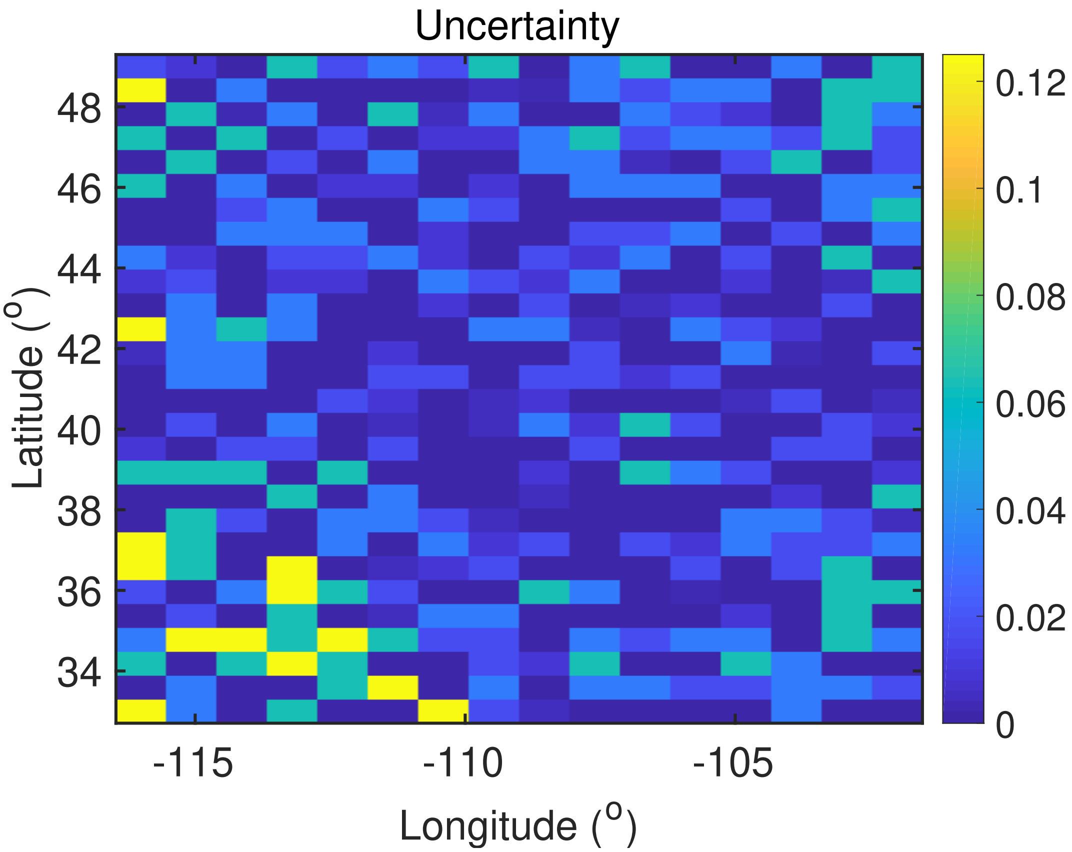

**Supplementary Figure 11.** Uncertainty map at the time slice of $t = 1435s$.

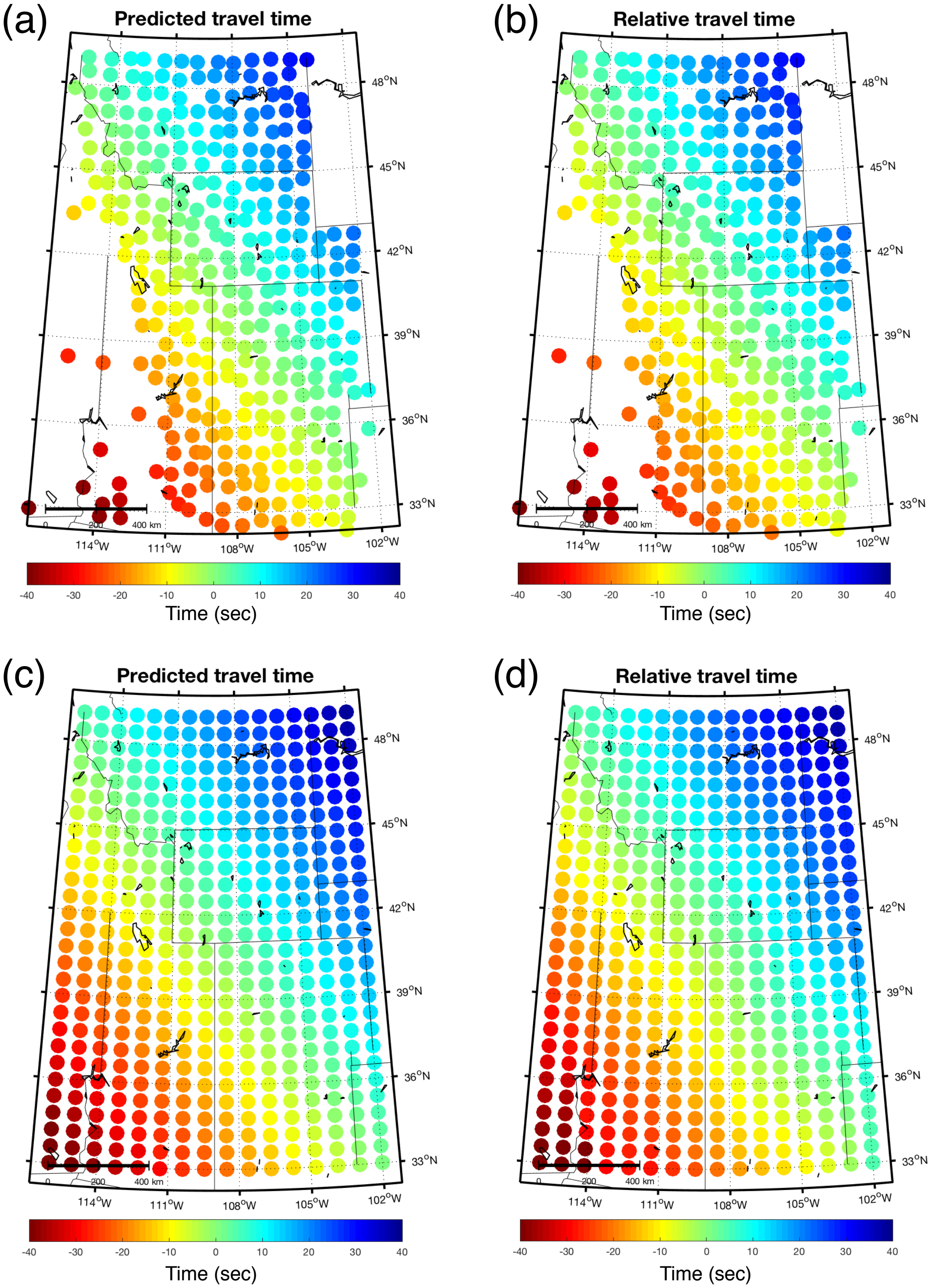


**Supplementary Figure 12.** Travel time measurements. (a) Predicted travel times at the original station locations based on the reference model. (b) Relative travel times of the original data based on cross-correlation measurements. (c) and (d) The same as (a) and (b) but for the reconstructed data.

## Supplementary References

1. Fomel, S. Shaping regularization in geophysical-estimation problems. Geophysics 72, R29–R36 (2007).
